# Prevalence and Serotype Diversity of *Salmonella* in Apparently Healthy Cattle: Systematic Review and Meta-Analysis of Published Studies, 2000–2017

**DOI:** 10.3389/fvets.2019.00102

**Published:** 2019-04-09

**Authors:** Fanta D. Gutema, Getahun E. Agga, Reta D. Abdi, Lieven De Zutter, Luc Duchateau, Sarah Gabriël

**Affiliations:** ^1^Department of Microbiology, Immunology and Veterinary Public Health, College of Veterinary Medicine and Agriculture, Addis Ababa University, Bishoftu, Ethiopia; ^2^Department of Veterinary Public Health and Food Safety, Faculty of Veterinary Medicine, Ghent University, Merelbeke, Belgium; ^3^Food Animal Environmental Systems Research Unit, United States Department of Agriculture, Agricultural Research Service, Bowling Green, KY, United States; ^4^Department of Veterinary Biomedical Sciences, College of Veterinary Medicine, Long Island University, Greenvale, NY, United States; ^5^Department of Nutrition, Genetics and Ethology, Faculty of Veterinary Medicine, Ghent University, Merelbeke, Belgium

**Keywords:** *Salmonella*, cattle, prevalence, serotypes, systematic review, meta-analysis

## Abstract

Salmonellosis is a leading cause of foodborne illnesses in humans with cattle being one of the reservoirs for *Salmonella*. We estimated a pooled prevalence of *Salmonella* in apparently healthy cattle and examined serotype diversity through systematic review and meta-analysis of studies published between 2000 and 2017. Peer reviewed publications reporting the prevalence of *Salmonella* in cattle were searched through five electronic databases (PubMed, Google scholar, Agricola, Scopus, CAB direct) and through manual search. We obtained 71 publications with 75 datasets consisting a total of 52,766 animals examined and 5,010 *Salmonella* positive cattle from 29 countries in six continents (except from Antarctica). Pooled prevalence of *Salmonella* in cattle was 9% (95% confidence interval: 7–11%). Significantly high heterogeneity (*I*^2^ = 98.7%, *P* < 0.01) was observed among all studies as well as within continents. Prevalence varied from 2% (Europe) to 16% (North America). Overall, 143 different serotypes were reported with the most diverse serotypes being reported from Africa (76 different serotypes) followed by North America (49 serotypes). The 10 most frequently reported serotypes (Montevideo, Typhimurium, Kentucky, Meleagridis, Anatum, Cerro, Mbandaka, Muenster, Newport, and Senftenberg) accounted for 65% of the isolates for which specific serotype information was reported. *Salmonella* Montevideo and *S*. Dublin are the most frequently reported serotypes in North America and Europe, respectively, while *S*. Typhimurium was the most frequent in Africa, Asia and Australasia. Our results indicated variability both in the prevalence and serotype diversity of *Salmonella* in cattle across continents. Although all *Salmonella* serotypes are potentially pathogenic to humans, five (Montevideo, Typhimurium, Anatum, Mbandaka, and Newport) of the top 10 serotypes identified in this study are among the serotypes most commonly associated with clinical illnesses in humans.

## Background

Foodborne illnesses pose public health and economic burdens both in developed and developing countries (1, 2). Annually, foodborne illnesses are responsible for an estimated 600 million cases, 420,000 deaths, and 33 million disability adjusted life years lost worldwide. *Salmonella* is a major cause of foodborne illnesses in humans (3–5). *Salmonella* are Gram-negative, non-spore forming, mostly motile, facultative anaerobic bacilli within the family *Enterobacteriaceae*. The species *Salmonella enterica* consists of six subspecies and more than 2,579 serovars (6, 7). Based on the clinical profiles of infections caused in humans *S. enterica* can be divided into typhoidal—which are human specific—and non-typhoidal *Salmonella* (NTS)—having a broad host range (6). The NTS serotypes are leading causes of bacterial diarrhea and invasive bacterial infections in young children, the elderly and the immune-compromised individuals throughout the world. *Salmonella* Typhimurium and *S*. Enteritidis together account for approximately 50% of all isolates globally reported from human clinical cases (8–10). The global incidence of diarrheal disease due to the NTS accounts for about 94 million enteric infections each year, of which 80.3 million cases are considered foodborne and resulting in 155,000 human deaths annually (11). Human salmonellosis is also recognized as an important socioeconomic disease posing considerable economic burden in the world (12, 13).

*Salmonella* colonizes mainly the intestinal tracts of humans and animals including cattle. Foods of animal origin are important sources of *Salmonella* infections in humans (13–18). Humans acquire the infection mainly through consumption of contaminated products including beef and beef products (19), by direct contact with infected animals or their environment (20) and by direct human-to-human transmission (21). Carcass contamination with *Salmonella* during slaughter, particularly under unsatisfactory hygienic operations, poses a significant public health risk (22–25). The transfer of NTS to food processing plants and equipment used for food preparation also plays an important role, ultimately leading to the risk of salmonellosis after the consumption of contaminated foods (21). Knowledge about the overall occurrence of *Salmonella* and the diversity of serotypes in cattle provides important information for decision making and to promote reliable efforts toward prevention and control of foodborne salmonellosis associated with cattle. Therefore, the objectives of this study were to determine the prevalence of *Salmonella* in apparently healthy cattle, and to assess the diversity of *Salmonella* serotypes associated with cattle production systems through a systematic review and meta-analysis of peer-reviewed publications between 2000 and 2017.

## Methods

### Systematic Review of the Literature

Preferred reporting items for systematic reviews and meta-analysis protocols (PRISMA-P) 2015 checklist was followed for the systematic review and meta-analysis of studies reporting *Salmonella* serotypes and prevalence in cattle (26). Five electronic databases were searched: PubMed (http://www.ncbi.nlm.nih.gov/pubmed), Google scholars (https://scholar.google.com/), Agricola (http://agricola.nal.usda.gov/), Scopus (http://www.scopus.com/), and CAB direct (http://www.cabdirect.org/). Additional publications were obtained by the manual scanning of the reference list from the retrieved publications. Salmonella, cattle, and prevalence were the main key words used for the search. The search was conducted with alternative terms for each key term using the general protocol [*Salmonella* AND (cattle OR bovine OR heifer OR bull OR bullock OR ruminant OR steer OR cow OR cull OR calf OR calves OR yearling OR beef OR dairy OR feedlot) AND (prevalence OR isolation OR identification OR “antimicrobial resistance” OR “antimicrobial susceptibility”)], that was modified and tailored to search strategies of each database when needed.

### Relevance Screening

The retrieved articles were imported to Refworks to manage and exclude duplicated studies (27). The duplicated records were excluded manually after making the bibliography list and prior to the eligibility assessment. The eligibility criteria were: (i) articles published in English between January 1, 2000 (since full articles could not be available online, publications prior to 2000 were not considered) and January 4, 2017 (the last date of literature search); (ii) reported on apparently healthy cattle (no statement is given about the inclusion of sick/diseased animals) from different production categories (dairy, beef, mixed) and sample sources (slaughter plant/abattoir/slaughter house, dairy farm, beef farm, ranch, feedlot, grazing point, market place, mixed cattle farm); (iii) samples collected from the intestinal content (feces from the rectum and other intestinal contents); (iv) prevalence report from any part of the world; and (v) cross-sectional study in which animal level prevalence was reported or could be calculated from the information provided in the publication during data extraction. The exclusion criteria were: (i) irrelevant records to the objective of the review; (ii) articles on sick or diseased cattle; (iii) non-cross-sectional study design; (iv) report on inappropriate samples such as ground or pen fecal or pooled fecal samples from which animal level prevalence was unknown, lymph nodes, rumen contents or other body parts of cattle; (v) when only citations or abstracts were available.

### Data Extraction

A peer-reviewed publication that describes prevalence of *Salmonella* in cattle was considered as a study unit. Cattle were considered positive for *Salmonella* when samples from the intestinal contents were tested and confirmed positive. When different prevalence reports in the content from various sites of intestinal tract were observed in a single study, we considered this one with the highest proportion for better precision to minimize under estimation. From each eligible publication, we extracted the following information: author, year of publication, year of study, study location (country and continent), detection method, production type (beef, dairy, and mixed), sampling location (abattoir, farm, market, ranch, grazing points, feedlot), age (calves and adults), amount of tested samples, sample size, number of *Salmonella* positive samples and serotypes identified, and number within each serotype. The extracted information was entered to a Microsoft excel spread sheet for quality assessment and data preparation for analysis.

### Data Analysis

Frequency distributions were used to describe the characteristics of the eligible publications and the diversity and proportion of *Salmonella* serotypes. Meta-analysis was conducted using the metaprop-one package (28), a Stata based program specifically designed for binominal data, that allows the computation of studies with 0 or 100% prevalence. Analysis was done in STATA version 14 (29). The prevalence of *Salmonella* in cattle was defined as the proportion of *Salmonella* positives based on the intestinal content samples. The pooled prevalence of *Salmonella* was computed by meta-analysis from the prevalence values of the individual publications by accounting for potential heterogeneity between studies and weighted on sample size (30). A logistic-normal random-effects model was used to model the within-study variability. The 95% confidence intervals (CIs) for the proportion of cattle *Salmonella* positive for the separate publications and their pooled prevalence was computed with the exact binomial method with the Freeman-Tukey double arcsine transformation which gives the CIs within admissible values. Further analysis of sub-groups of the overall estimate was performed according to age, production type, detection method, and continent categories. Heterogeneity of the effect sizes among the publications was assessed by Cochrane Q test and inverse variance index (I^2^) test and quantified as recommended by Higgins and Thompson (31). A *P* < 0.01 was set as an indication of a statistically significant heterogeneity. The basic results from the meta-analysis were visually presented using forest plots. Frequency distributions were used to describe the characteristics of the eligible studies and the diversity and proportion of *Salmonella* serotypes.

## Results

### Systematic Review of the Literature

**Figure 1:**
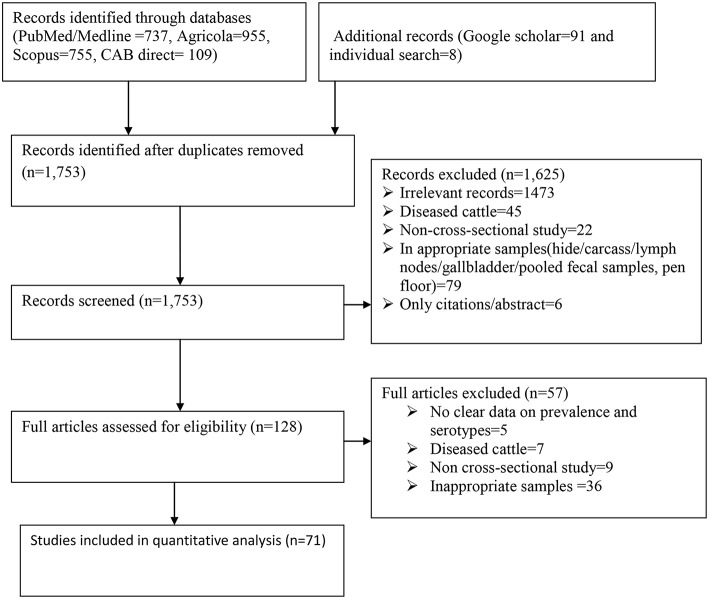
Flow diagram for the selection of studies included in the meta-analysis for the prevalence of *Salmonella* in apparently healthy cattle.

A flow chart showing the systematic literature search procedure is shown in Figure 1. A total of 2,655 records were retrieved from the five search engines (PubMed, Google scholar, Agricola, Scopus, and CAB direct) and by manual search. After de-duplicating the references, 1,753 publications were retained for further screening. After relevance screening of the titles and abstracts, 1,625 articles were excluded resulting in 128 potentially eligible full articles. Further in-depth eligibility assessment of the full articles resulted in 71 eligible publications for data extraction and analysis. The references of all the eligible articles are listed in Supplementary Table 1.

### Data Extraction and Meta-Analysis

Data were extracted from the 71 peer-reviewed publications comprising 75 data sets. Two separate datasets were extracted from three publications (32–34) based on age and from one study (35) based on sampling points. Therefore, 75 data sets (hereafter referred to as studies) comprising fecal samples or swabs from 52,766 animals were included in the meta-analysis. *Salmonella* was detected in 5,010 of the animals. Over two-thirds (68%) of the studies used ≤ 10 g of feces, and 91% of the studies used traditional culture methods for the detection of *Salmonella*. The publications represented 29 countries across six continents except Antarctica. While 80% of the countries were represented by one or two publications, the United States was the most represented with 25 publications. Forty percent of the studies were conducted on samples collected at processing plants. Characteristics of the publications are shown in Table 1.

**Table 1:** Description of the eligible publications included in the systematic review and meta-analysis of *Salmonella* in apparently healthy cattle.

**Characteristics**	**Number of datasets (*n* = 75)**	**Percentage**
**Fecal amount (g OR ml)**		
≤ 10	51	68.0
>10	7	9.3
Swabs/loopful	12	16.0
Not specified	5	6.7
**Sampling point**		
Dairy farm	25	33.3
Abattoir	30	40.0
Feedlot	7	9.3
Grazing point	2	2.7
Mixed farm	8	10.7
Not specified	2	2.7
Market	1	1.3
**Detection methods**		
Traditional culturing	68	90.7
IMS	6	8.0
PCR	1	1.3
**Age**		
Adult	63	84.0
Calves	12	16.0
**Production type**		
Beef	18	24.0
Dairy	28	37.3
Mixed	14	18.7
Not specified	15	20.0
**Continent**		
Africa	16	21.3
Asia	15	20.0
Australasia	6	8.0
Europe	9	12.0
North America	28	37.3
South America	1	1.3

*IMS, Immunomagnetic separation; PCR, Polymerase chain reaction*.

**Figure 2:**
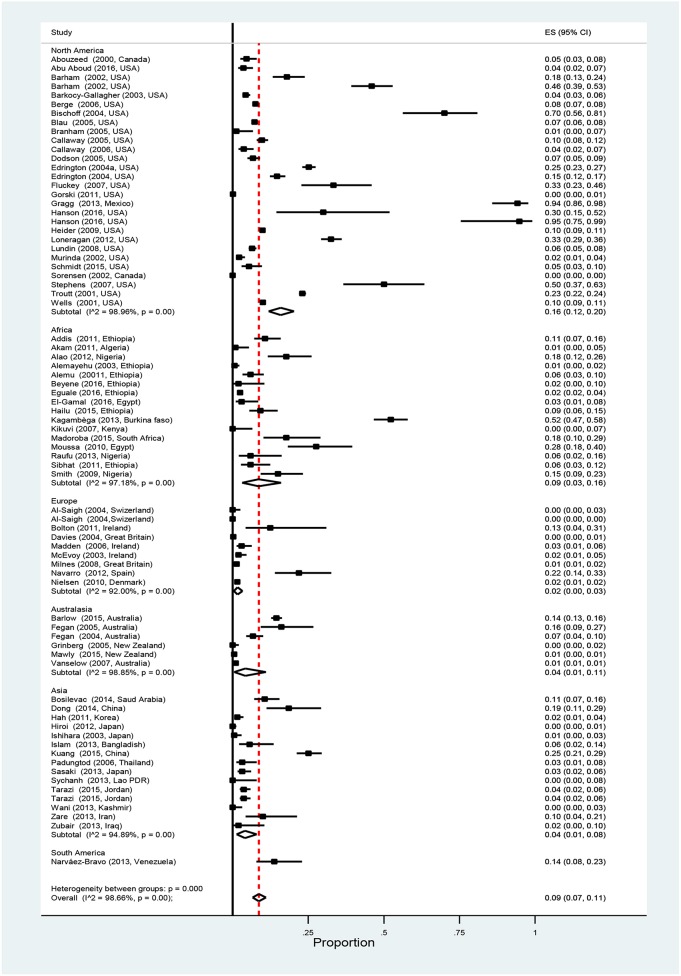
Forest plot showing estimated individual and overall *Salmonella* prevalence in apparently healthy cattle (ES, effect size; *CI, confidence interval; I*^2^, *Inverse variance index)*.

Overall pooled prevalence of *Salmonella* in cattle was 9% (95% CI: 7–11%). Results of individual studies along with the effect of sizes are shown in Figure 2. Study prevalence values ranged from 0 to 95%. Test of heterogeneity demonstrated the presence of a high degree of heterogeneity (*I*^2^ = 98.7%, *P* < 0.01) among the studies. To account for some of the variability separate stratified meta-analyses were performed by age, production type, detection method, and continent (Table 2). The pooled prevalence of *Salmonella* is higher in the adult cattle [9% (95% CI: 7–12%)] than in the calves [6% (95% CI: 2–11%)], in beef cattle [14% (95% CI: 7–23%)] than in other production types, and in North America [16% (95% CI:12–20%)] than in other continents. Studies within each category of the strata defined by detection method and continent, showed significantly high degrees of heterogeneities (*P* < 0.01). However, no significant heterogeneity was observed between the age groups, among production types and when comparing only between immunomagnetic separation (IMS) and non-IMS detection methods (*P* > 0.01).

**Table 2:** Pooled prevalence of *Salmonella* in apparently healthy cattle determined by meta-analysis of 75 datasets studies by age, production type, detection method, and continent.

**Subgroups**	**No. of publications**	**No. of datasets**	**No. of animals tested**	**No. of animals positive**	**Pooled prevalence (95% confidence interval)**	**Heterogeneity test**	
						*I*^2^*^^ (%)	***p-value***
**AGE**							
Adult	62	63	45,289	4,624	9 (7-12)	98.7	<0.01
Calves	12	12	7,477	386	6 (2-11)	97.4	<0.01
**PRODUCTION TYPE**							
Beef	17	18	5,085	366	14 (7-23)	98.3	<0.01
Dairy	26	28	30,970	3,746	10 (7-13)	98.7	<0.01
Mixed	13	14	10,154	588	5 (2-9)	98.0	<0.01
Not specified	15	15	6,557	310	5 (2-11)	97.9	<0.01
**DETECTION METHOD**							
Non-IMS	64	68	50,311	4,696	8 (6-11)	98.7	<0.01
PCR	1	1	50	25	50 (37-63)	-	-
IMS	6	6	2,405	289	10 (5-16)	92.1	<0.01
**CONTINENT**							
Africa	16	16	3,153	314	9 (3-16)	98.2	<0.01
Asia	14	15	3,116	202	4 (1-8)	94.9	<0.01
Australasia	6	6	6,370	287	4 (1-11)	98.8	<0.01
Europe	8	9	6,470	88	2 (0–3)	92.0	<0.01
North America	26	28	33,577	4,108	16 (12-20)	99.0	<0.01
South America	1	1	80	11	14 (8-23)	–	–
**Total**	71	75	52,766	5,010	9 (7-11)	98.7	<0.01

* *Inverse variance index that describes the percentage of variation across studies attributed to heterogeneity rather than chance*.

### Diversity of Serotypes

Serotype information was not reported for 1,926 *Salmonella* positive cattle from a total of 16,175 cattle examined in 27 publications representing 29 data sets. In the remaining 44 publications representing 46 datasets for which serotype information was available, 3,191 *Salmonella* isolates were reported from 3,084 *Salmonella* positive cattle from a total of 36,591 cattle examined. Among the 3,191 isolates with serotyping information, specific serotypes were reported in 91.6% (2,923/3,191) of the isolates while 2.8% of the isolates were untypable, and the remaining 5.6% were reported as “other serotypes” where the list of which was not stated in the publication.

Overall, 143 different serotypes were reported among the 2,923 *Salmonella* isolates listed in the data sets included in the meta-analysis. The most frequently (with ≥1%) reported serotypes are shown in Table 3 and the list of serotypes (<1%) categorized as “others” in the latter table is presented in the Supplementary Table 2. The 10 most frequently reported cattle associated serotypes across all studies were *S*. Montevideo, Typhimurium, Kentucky, Meleagridis, Anatum, Cerro, Mbandaka, Muenster, Newport, and Senftenberg. These 10 most frequently isolated serotypes comprised 69.5% (2,032/2,923) of total isolates for which specific serotypes were reported. There were variations in the frequency and diversity of *Salmonella* serotypes in the six continents for which publications were retrieved (Table 4). *S*. Montevideo was the most frequent reported serotype from North America, while this serotype did not belong to the five most frequently reported serotypes in most other continents. *Salmonella* Typhimurium was the most frequently reported serotype in Africa, Asia, and Australasia, while *S*. Dublin was the most frequently reported serotype in Europe. The most diverse serotypes were reported from Africa (76 different serotypes) followed by North America (49 different serotypes), Australasia (39 serotypes), Asia (23 serotypes), Europe (12 serotypes), and South America (2 serotypes).

**Table 3:** *Salmonella* isolates by serotype in descending order of frequency across studies reporting specific serotypes.

**Serotypes**	**No. of isolates**	**Percentage (*n* = 2,923)**	**No. of datasets (%)**	**Continent (number of isolates representing each serotype)**
Montevideo	524	17.9	14 (30.4)	Africa (1), Asia (6), Australasia (2), North America (515)
Typhimurium	294	10.1	28 (60.9)	Africa (45), Asia (49), Australasia (96), Europe (12), North America (91)
Kentucky	214	7.3	11 (23.9)	Africa (5), Asia (1), North America (208)
Meleagridis	186	6.4	11 (23.9)	Asia (5), Australasia (2), Europe (4), North America (175)
Anatum	179	6.1	17 (36.9)	Africa (2), Asia (7), Australasia (24), Europe (10), North America (136)
Cerro	176	6.0	7 (15.2)	Australasia (3), North America (173)
Mbandaka	169	5.8	12 (26.1)	Australasia (6), Europe (10), North America (153)
Muenster	113	3.9	6 (13)	Africa (17), North America (96)
Newport	92	3.1	10 (21.7)	Africa (3), Australasia (1), North America (86)
Senftenberg	85	2.9	9 (19.6)	Asia (4), Australasia (9), North America (72)
Dublin	64	2.2	10 (21.7)	Africa (6), Australasia (9), Europe (38), North America (11)
Agona	62	2.1	13 (28.3)	Asia (21), Australasia (3), North America (38)
Menhaden	59	2.0	1 (2.2)	North America (59)
Muenchen	53	1.8	5 (10.9)	North America (47), Australasia (6)
Infantis	51	1.7	1 (2.2)	North America (51)
Give	47	1.6	1 (2.2)	Australasia (47)
Others^#^	555	18.9.1		

*Data are from 46 datasets that reported serotype information*;

# See Supplementary Table 2 for the list of serotypes categorized as “others.”

**Table 4:** *Salmonella* isolates by serotype within six continents in descending order of frequency in studies reporting specific serotypes.

**Rank**	Serotypes (% of isolates)^*^					
	**North America**	**Africa**	**Asia**	**Australasia**	**Europe**	**South America**
1	Montevideo (24.0)	Typhimurium (15.8)	Typhimurium (40.0)	Typhimurium (34.4)	Dublin (44.7)	Javiana (50.0)
2	Kentucky (9.7)	Drac (26, 9.1)	Agona (17)	Anatum (8.6)	Typhimurium (14.1)	Weltevreden (50.0)
3	Meleagridis (8.2)	Enteritidis (8)	Derby (6.5)	Orion (6.8)	Anatum (11.8)	
4	Cerro (8.1)	Muenster (5.9)	Anatum (5.6)	Bovismorbificans (6.1)	Mbandaka (11.8)	
5	Mbandaka (7.1)	Bredeney (5.6)	Montevideo (4.8)	Saintpaul (5.4)	Derby (4.7)	
6	Anatum (6.3)	Urbana (4.5)	Meleagridis (4.0)	Dublin (3.2)	Meleagridis (4.7)	
7	Muenster (4.5)	Ruiru (2.8)	Enteritidis (3.2)	Zanzibar (3.2)	London [10+]	
8	Typhimurium (4.2)	Dublin (2.1)	Kunduchi (3.2)	Infantis (2.9)	6,7: D: - (1.2)	
9	Newport (4.0)	Saintpaul (2.1)	Senftenberg (3.2)	Thompson (2.5)	Agama (1.2)	
10	Senftenberg (3.4)	Virchow (2.1)	Fyris (1.6)	Havana (2.5)	Kedougou (1.2)	
11	Menhaden (2.8)	Hato (1.8)	Kingston (1.6)	Senftenberg (2.5)	Kiel (1.2)	
12	Muenchen (2.2)	Kentucky (1.8)	Rissen (1.6)	Mbandaka (2.2)	Othmarschen (1.2)	
13	Give (2.1)	Newport (1.8)		Muenchen (2.2)		
14	Infantis (1.9)	Tennessee (1.8)		Bredeney (1.8)		
15	Agona (1.8)	Chomedey (1.4)		Adelaide (1.4)		
16	Minnesota (1.4)	Lagos (1.4)		Chester (1.4)		
17	Kinshasa (1.0)	Soumbedioune (1.4)		Agona (1.1)		
18		Eko (1.1)		Cerro (1.1)		
19		Farakan (1.1)		Charity (1.1)		
20		Mishmarhaemek (1.1)		Ruiru (1.1)		
21		Nima (1.1)				
22		Uganda (1.1)				
Other	32 serotypes (7.5)	55 serotypes (19.3)	9 serotypes (7.3)	19 serotypes (8.6)	–	–
Total no.	2,148	285	124	279	85	2

* *only serotypes with ≥1% frequency are reported, and the rest are categorized as other*.

## Discussion

To the best of our knowledge, this is the first estimate of the overall *Salmonella* prevalence and the diversity of serotypes in apparently healthy cattle. We used a systematic method to identify articles reporting the prevalence of *Salmonella* and the serotypes in such cattle, followed by a quantitative meta-analysis to estimate the overall prevalence of *Salmonella* at the global level.

*Salmonella* colonizes the gastrointestinal tract of food animals (7) and is shed via feces (36–39). Cattle are asymptomatic carriers or reservoirs for *Salmonella* and may function as a source of foodborne infection (8, 23, 24). A number of serotypes frequently isolated from humans have been isolated from sick or asymptomatic cattle and some human cases have also been linked to direct exposure to cattle (20). Knowing the prevalence and diversity of *Salmonella* serotypes in cattle can provide important information necessary to develop preventive measures and strategies at different stages of the food chain such as application hazard analysis and critical control point (HACCP) programs in beef and milk production industries to ensure food safety (40).

There was high heterogeneity in the estimated *Salmonella* prevalence among the studies included in the analysis. The *Salmonella* prevalence can vary depending on the detection method used, the amount of sample processed, production type, and geographical variation in the distribution of the *Salmonella* (32, 41). The overall pooled prevalence of 9% is higher compared to other reported national level prevalence values ranging from 0.2 to 7.1% (42–46). This is not surprising since our meta-analysis provides a precise estimate (with narrow confidence interval) as it includes a higher amount of samples and total number of positive cattle for *Salmonella* by pooling 75 datasets from 71 publications.

The prevalence was higher in the adult cattle [9% (95%CI: 7–12%)] than in the young age group [6%(95%CI: 2–11%)]. Although the effect of age needs further investigation, this variation can presumably be in part due to variation in the number of studies included in the meta-analysis in each age group. In the young age group there were 12 publications representing only 14.2% (*n* = 7,477) of total cattle examined compared to 63 publications in the adult cattle with 86% of the total cattle examined. Over 70% of the publications were conducted at processing plants and in culled dairy cows destined for slaughter perhaps because of the higher public health significance at the final stage of production chain that is close to consumers (47). Even though *Salmonella* colonizes the intestinal tracts of cattle, there is no difference in the colonization and shedding of *Salmonella* between healthy calves and adult cattle (7). However, a higher prevalence of *Salmonella* shedding animals occurs when asymptomatic chronically infected carrier cattle are present on the farm and stay on the farm for long periods (45), which may contribute to transmission and persistence of *Salmonella* on the farm.

Although not statistically significant, the prevalence was higher in beef cattle compared to dairy cattle. This apparent difference can be attributed to how the animals were sampled. In most of the studies culled dairy cows were sampled at farms before shipment as opposed to beef cattle which were commonly sampled at the processing plants. Temporary restriction or complete feed withdrawal (48) and exposure to stress such as transport (42, 49, 50) can result in increased fecal shedding of *Salmonella* in feedlot cattle prior to slaughter.

Variations in prevalence that ranged from 2% (Europe) to 16% (North America) in various continents of the world could partly be attributed to the differences in the number of publications and the number of cattle samples included in the analysis. For North America, 26 publications (28 data sets) were retrieved consisting of 33,577 cattle samples, being the majority of the articles. In contrast, the very low prevalence estimate (2%) observed in Europe, was estimated only from 8 publications (9 data sets) in which 6,470 cattle sampled. The prevalence in South America was 14%, however this does not represent the pooled estimate as only one article was included in the analysis. The differences might also be associated with the differences in the monitoring and surveillance mechanisms among the continents (51).

Difference in the prevalence was also observed among categories of detection methods. In the majority (91%) of the studies, *Salmonella* was detected using traditional culturing methods which are in general considered less sensitive methods. Limited number of studies used immunomagnetic separation beads or PCR. Moreover, variation in the sensitivity of culture detection methods can influence the prevalence and consequently the observed heterogeneity (52).

In this systematic review, *S*. Montevideo and *S*. Typhimurium were the two most frequent and dominant serotypes reported where *S*. Montevideo was majorly reported from North America. *Salmonella* Typhimurium is one of the major serotypes that accounted for human clinical cases globally (10). Human infections and outbreaks due to *S*. Montevideo is also increasing around the globe (53)and reported in the USA, Europe, Australia, and Asia (54–56). There were differences in the most commonly reported serotypes and their proportions among different continents. *Salmonella* Typhimurium which is historically associated with cattle ranked number one in Africa, Asia, and Australasia. In North America and Europe, however, *S*. Montevideo and *S*. Dublin ranked number one, respectively. The implication of the shift in serotype with respect to public health requires further study. Interestingly, among the top 10 *Salmonella* serotypes identified in this study, *S*. Montevideo, *S*. Typhimurium, *S*. Anatum, *S*. Mbandaka, and *S*. Newport are among the World Health Organization's top 20 serotypes associated with human salmonellosis across the world (52). Spatial and temporal effects on the distribution and diversity of *Salmonella* have been reported (57, 58), which may explain the observed differences in the serotype diversity among the studies reporting *Salmonella*. Some of the serotypes reported in the present review were identified as the dominant serotypes elsewhere in cattle at varying proportions. For instance, in the USA, *S*. Newport (48.7%) and *S*. Typhimurium (7.1%) (59); in Ethiopia, *S*. Typhimurium (17.4%), *S*. Newport (13%) and *S*. Anatum (5.8%) (42), and in Europe, *S*. Typhimurium (38.6%) were reported to be the most frequent and dominant serotypes (60). On the contrary, none of these serotypes were reported from the national survey of *Salmonella* serotypes in cattle carried out in Japan (41).

All non-typhoidal *Salmonella* serotypes except a few serotypes which are host-specific, can potentially cause disease in humans and reside in one or more animal species (61). *Salmonella* serotypes were reported to be linked to several outbreaks following the consumption of contaminated beef, milk, and products thereof (62). *S*. Enteritidis and *S*. Typhimurium are the two most important serotypes transmitted from animals to humans in most parts of the world (51, 60, 63, 64). In the USA, 29 cases of diarrheal illness caused by *S*. Typhimurium were associated with the consumption of raw milk or raw-milk products from dairy cattle (65). During the period 1973–2011, of the 1,965 *Salmonella* outbreaks where a food vehicle was implicated, 96 were attributed to beef, accounting for 3,684 illnesses in USA. *S*. Newport and *S*. Typhimurium accounted for 18 and 17% of illnesses, and 29 and 18% of hospitalizations, respectively (19). The multidrug-resistant *S*. Typhimurium DT104 has also been associated with outbreaks related to beef contamination and resulted in hospitalization rates twice as that of other foodborne salmonellosis cases (65). From a total of 1,168 foodborne outbreaks of human salmonellosis in 2013 reported by the European member states, 1.6% of the cases were attributed to beef and beef products (60). This systematic review showed that *S*. Typhimurium was the most frequently reported serotype from cattle in Africa, Asia, and Australasia. Cattle could also contribute to the invasive non-typhoidal *Salmonella* disease in people who have contact with cattle feces. This is particularly important in regions like Africa where invasive non-typhoidal *Salmonella* infections are endemic as reviewed by Marks et al. (66). All the above evidence supports the importance of cattle and cattle associated serotypes for human salmonellosis.

Besides the datasets from the publications included in this review and meta-analysis, other relevant information was available in new articles that were published in the years 2017 and 2018 while the manuscript was under preparation by the authors. During this period, 6 full articles and three published abstracts representing 11 datasets were retrieved using the search engines (67–75). The majority of these studies were reported from Africa (67, 68, 70–74) except for two studies from Europe (69) and South America (75). Among the total of 5,868 cattle examined, 9.2% (554/6018), which is nearly equal to the pooled prevalence estimate, were reported to be positive for *Salmonella* species with different serotypes. The global level pooled prevalence of *Salmonella* in cattle was higher (9%) as compared to the pooled prevalence estimates of *E. coli* O157 (5.68%), which is also excreted by cattle showing the relative public health importance of *Salmonella* (76).

## Conclusions

This study based on systematic reviews and meta-analysis provides an overall prevalence of *Salmonella* and serotype diversity in apparently healthy cattle at a global level. The results indicated variations in the level of *Salmonella* carriage in cattle across the world, and the presence of a diverse number of *Salmonella* serotypes. The estimated *Salmonella* prevalence was higher in North America. The predominant detection method is traditional culturing. Because of the possibility of *Salmonella* contamination of carcasses during slaughter and milk during milking, cattle can be a potential source of *Salmonella* and can lead to public health risk and economic loss if the necessary hygienic measures are not properly followed.

## Author Contributions

FG, GA, RA, LD, and LDZ designed the study and identified the search engines and key words for literature search. FG, GA, and LD analyzed the data. FG wrote the manuscript. GA, RA, LD, LDZ, and SG revised the manuscript.

### Conflict of Interest Statement

The authors declare that the research was conducted in the absence of any commercial or financial relationships that could be construed as a potential conflict of interest.
