# Corrigendum: Prevalence and Serotype Diversity of *Salmonella* in Apparently Healthy Cattle: Systematic Review and Meta-Analysis of Published Studies, 2000–2017

**DOI:** 10.3389/fvets.2019.00184

**Published:** 2019-06-11

**Authors:** Fanta D. Gutema, Getahun E. Agga, Reta D. Abdi, Lieven De Zutter, Luc Duchateau, Sarah Gabriël

**Affiliations:** ^1^Department of Microbiology, Immunology and Veterinary Public Health, College of Veterinary Medicine and Agriculture, Addis Ababa University, Bishoftu, Ethiopia; ^2^Department of Veterinary Public Health and Food Safety, Faculty of Veterinary Medicine, Ghent University, Merelbeke, Belgium; ^3^Food Animal Environmental Systems Research Unit, United States Department of Agriculture, Agricultural Research Service, Bowling Green, KY, United States; ^4^Department of Veterinary Biomedical Sciences, College of Veterinary Medicine, Long Island University, Greenvale, NY, United States; ^5^Department of Nutrition, Genetics and Ethology, Faculty of Veterinary Medicine, Ghent University, Merelbeke, Belgium

**Keywords:** *Salmonella*, cattle, prevalence, serotypes, systematic review, meta-analysis

In the original article, there was an error. The article cited as (69) referred to “Africa,” and should have referred to “Europe.”

A correction has been made to the **Discussion**, paragraph 10:

“Besides the datasets from the publications included in this review and meta-analysis, other relevant information was available in new articles that were published in the years 2017 and 2018 while the manuscript was under preparation by the authors. During this period, 6 full articles and three published abstracts representing 11 datasets were retrieved using the search engines (67–75). The majority of these studies were reported from Africa (67, 68, 70–74) except for two studies from Europe (69) and South America (75). Among the total of 5,868 cattle examined, 9.2% (554 /6018), which is nearly equal to the pooled prevalence estimate, were reported to be positive for *Salmonella* species with different serotypes. The global level pooled prevalence of *Salmonella* in cattle was higher (9%) as compared to the pooled prevalence estimates of *E. coli* O157 (5.68%), which is also excreted by cattle showing the relative public health importance of *Salmonella* (76).”

The authors apologize for this error and state that this does not change the scientific conclusions of the article in any way. The original article has been updated.

