# Supplementary material for: Prevalence and Serotype Diversity of Salmonella in Apparently Healthy Cattle: Systematic Review and Meta-Analysis of Published Studies, 2000–2017
Source: Front Vet Sci. 2019 Apr 9;6:102. doi: 10.3389/fvets.2019.00102 (PMC6476277; doi:10.3389/fvets.2019.00102)
Supplement: Supplementary file 1 [file Table_1.docx]

Supplementary Table 1: List of studies included in the meta-analysis

1. Abouzeed YM, Hariharan H, Poppe C, Kibenge FS. Characterization of *Salmonella* isolates from beef cattle, broiler chickens and human sources on Prince Edward Island. Comparative Immunology, Microbiology and Infectious Diseases. 2000; 23(4):253-66.
2. Troutt HF, Galland JC, Osburn BI, Brewer RL, Braun RK, Schmitz JA, Sears P, Childers AB, Richey E, Mather E, Gibson M. Prevalence of *Salmonella* spp in cull (market) dairy cows at slaughter. Journal of the American Veterinary Medical Association. 2001; 219(9):1212-5.
3. Wells SJ, Fedorka-Cray PJ, Dargatz DA, Ferris K, Green A. Fecal shedding of *Salmonella* spp. by dairy cows on farm and at cull cow markets. Journal of Food Protection. 2001; 64(1):3-11.
4. Barham AR, Barham BL, Johnson AK, Allen DM, Blanton Jr JR, Miller MF. Effects of the transportation of beef cattle from the feed yard to the packing plant on prevalence levels of *Escherichia coli O157* and *Salmonella* spp. Journal of Food Protection. 2002; 65 (2):280-3.
5. Murinda SE, Nguyen LT, Ivey SJ, Gillespie BE, Almeida RA, Draughon FA, Oliver SP. Molecular characterization of *Salmonell*a spp. isolated from bulk tank milk and cull dairy cow fecal samples. Journal of Food Protection. 2002; 65(7):1100-.
6. Sorensen O, VanDonkersgoed JO, McFALL MA, Manninen K, Gensler G, Ollis G. Salmonella spp. shedding by Alberta beef cattle and the detection of *Salmonella* spp. in ground beef. Journal of Food Protection. 2002; 65(3):484-91.
7. Alemayehu D, Molla B, Muckle A.Prevalence and antimicrobial resistance of *Salmonella* isolated from apparently healthy slaughtered cattle in Ethiopia. Tropical Animal Health Production.2003; 35: 309-316.
8. Barkocy-Gallagher GA, Arthur TM, Rivera-Betancourt M, Nou X, Shackelford SD, Wheeler TL, Koohmaraie M. Seasonal prevalence of Shiga toxin–producing *Escherichia coli*, including O157: H7 and non-O157 serotypes, and *Salmonella* in commercial beef processing plants. Journal of Food Protection. 2003; 66(11):1978-86.
9. Ishihara K, Takahashi T, Morioka A, Kojima A, Kijima M, Asai T, Tamura Y. National surveillance of *Salmonella enterica* in food-producing animals in Japan. Acta Veterinaria Scandinavica. 2009: 51(1):35
10. McEvoy JM, Doherty AM, Sheridan JJ, Blair IS, McDowell DA. The prevalence of *Salmonella* spp. in bovine faecal, rumen and carcass samples at a commercial abattoir. Journal of Applied Microbiology. 2003; 94(4):693-700.
11. Al-Saigh H, Zweifel C, Blanco J, Blanco JE, Blanco M, Usera MA, Stephan R. Fecal shedding of Escherichia coli O157, Salmonella, and Campylobacter in Swiss cattle at slaughter. Journal of Food Protection. 2004; 67(4):679-84.
12. Bischoff KM, Edrington TS, Callaway TR, Genovese KJ, Nisbet DJ. Characterization of antimicrobial resistant *Salmonella* Kinshasa from dairy calves in Texas. Letters in Applied Microbiology. 2004; 38(2):140-5.
13. Davies RH, Dalziel R, Gibbens JC, Wilesmith JW, Ryan JM, Evans SJ, Byrne C, Paiba GA, Pascoe SJ, Teale CJ. National survey for Salmonella in pigs, cattle and sheep at slaughter in Great Britain (1999–2000). Journal of Applied Microbiology. 2004; 96(4):750-60.
14. Edrington, TS. Schultz, CL, Bischoff, KM, Callaway, TR Looper, ML Genovese, KJ, Jung YS, Mcreynolds, JL, Anderson RC and Nisbet DJ.Antimicrobial resistance and serotype prevalence of *Salmonella* isolated from dairy cattle in the southwestern United States. Microbial Drug Resistance. 2004; 10(1):51-6.
15. Edrington TS, Hume ME, Looper ML, Schultz CL, Fitzgerald AC, Callaway TR, Genovese KJ, Bischoff KM, McReynolds JL, Anderson RC, Nisbet DJ. Variation in the faecal shedding of *Salmonell*a and *E. coli O157: H7* in lactating dairy cattle and examination of *Salmonella* genotypes using pulsed‐field gel electrophoresis. Letters in Applied Microbiology. 2004; 38(5):366-72.
16. Fegan N, Vanderlinde P, Higgs G, Desmarchelier P. Quantification and prevalence of *Salmonella* in beef cattle presenting at slaughter. Journal of Applied Microbiology. 2004; 97(5):892-8.
17. Blau DM, McCluskey BJ, Ladely SR, Dargatz DA, Fedorka-Cray PJ, Ferris KE, Headrick ML. *Salmonella* in dairy operations in the United States: prevalence and antimicrobial drug susceptibility. Journal of Food Protection. 2005;68(4):696-702.
18. Branham LA, Carr MA, Scott CB, Callaway TR. *E. coli O157* and *Salmonella* spp. in white-tailed deer and livestock. Current Issues in Intestinal Microbiology. 2005; 6(2):25-9.
19. Callaway TR, Keen JE, Edrington TS, Baumgard LH, Spicer L, Fonda ES, Griswold KE, Overton TR, VanAmburgh ME, Anderson RC, Genovese KJ. Fecal prevalence and diversity of *Salmonella* species in lactating dairy cattle in four states. Journal of Dairy Science. 2005; 88(10):3603-8.
20. Dodson K, Lejeune J. Escherichia coli O157: H7, *Campylobacter jejuni*, and *Salmonella* prevalence in cull dairy cows marketed in northeastern Ohio. Journal of Food Protection. 2005; 68(5):927-31.
21. Fegan N, Vanderlinde P, Higgs G, Desmarchelier P. A study of the prevalence and enumeration of *Salmonella enterica* in cattle and on carcasses during processing. Journal of Food Protection. 2005; 68(6):1147-53.
22. Grinberg A, Pomroy WE, Weston JF, Ayanegui-Alcerreca A, Knight D. The occurrence of *Cryptosporidium parvum, Campylobacter* and *Salmonella* in newborn dairy calves in the Manawatu region of New Zealand. New Zealand Veterinary Journal. 2005;53(5):315-20.
23. Berge AC, Moore DA, Sischo WM. Prevalence and antimicrobial resistance patterns of *Salmonella enterica* in preweaned calves from dairies and calf ranches. American Journal of Veterinary Research. 2006; 67(9):1580-8.
24. Callaway TR, Edrington TS, Brabban AD, Keen JE, Anderson RC, Rossman ML, Engler MJ, Genovese KJ, Gwartney BL, Reagan JO, Poole TL. Fecal prevalence of *Escherichia coli O157, Salmonella, Listeria*, and bacteriophage infecting *E. coli O157: H7* in feedlot cattle in the southern plains region of the United States. Foodborne Pathogens and Disease. 2006; 3(3):234-44.
25. Madden RH, Murray KA, Gilmour A. Carriage of four bacterial pathogens by beef cattle in Northern Ireland at time of slaughter. Letters in Applied Microbiology. 2007; 44(2):115-9.
26. Padungtod P, Kaneene JB*. Salmonella* in food animals and humans in northern Thailand. International Journal of Food Microbiology. 2006; 108(3):346-54.
27. Fluckey WM, Loneragan GH, Warner R, Brashears MM. Antimicrobial drug resistance of *Salmonella* and *Escherichia coli* isolates from cattle feces, hides, and carcasses. Journal of Food Protection. 2007; 70(3):551-6.
28. Kikuvi GM, Ombui JN, Mitema ES, Schwarz S. Antimicrobial resistance in Salmonella serotypes isolated from slaughter animals in Kenya. East African Medical Journal. 2007; 84(5):233-9.
29. Stephens TP, Loneragan GH, Thompson TW, Sridhara A, Branham LA, Pitchiah S, Brashears MM. Distribution of *Escherichia coli O157* and *Salmonella* on hide surfaces, the oral cavity, and in feces of feedlot cattle. Journal of Food Protection. 2007; 70(6):1346-9.
30. Vanselow BA, Hornitzky MA, Walker KH, Eamens GJ, Bailey GD, Gill PA, Coates K, Corney B, Cronin JP, Renilson S. Salmonella and on‐farm risk factors in healthy slaughter‐age cattle and sheep in eastern Australia. Australian Veterinary Journal. 2007;85(12):498-502.
31. Lundin JI, Dargatz DA, Wagner BA, Lombard JE, Hill AE, Ladely SR, Fedorka-Cray PJ. Antimicrobial drug resistance of fecal Escherichia coli and Salmonella spp. isolates from United States dairy cows. Foodborne Pathogens and Disease. 2008;5(1):7-19.
32. Milnes AS, Stewart I, Clifton-Hadley FA, Davies RH, Newell DG, Sayers AR, Cheasty T, Cassar C, Ridley A, Cook AJ, Evans SJ. Intestinal carriage of verocytotoxigenic *Escherichia coli O157, Salmonella, thermophilic Campylobacter* and *Yersinia enterocolitica*, in cattle, sheep and pigs at slaughter in Great Britain during 2003. Epidemiology and Infection. 2008; 136(6):739-51.
33. Heider LC, Funk JA, Hoet AE, Meiring RW, Gebreyes WA, Wittum TE. Identification of *Escherichia coli* and *Salmonella enterica* organisms with reduced susceptibility to ceftriaxone from fecal samples of cows in dairy herds. American Journal of Veterinary Research. 2009; 70(3):389-93.
34. Smith S, Bamidele M, Goodluck H, Fowora M, Omonigbehin E, Oper B, Aboaba O. Antimicrobial susceptibilities of Salmonellae isolated from food handlers and Cattle in Lagos, Nigeria. International Journal of Health Research. 2009; 2(2).
35. Moussa IM, Ashgan MH, Mohamed MS, Mohamed KH, Al-Doss AA. Rapid detection of *Salmonella* species in newborne calves by polymerase chain reaction. International Journal of Genetics and Molecular Biology. 2010; 2(4):062-6.
36. Nielsen LR, Baggesen DL, Aabo S, Moos MK, Rattenborg E. Prevalence and risk factors for *Salmonella* in veal calves at Danish cattle abattoirs. Epidemiology & Infection. 2011; 139(7):1075-80.
37. Hah DY, Ji DH, Jo SR, Park AR, Jung EH, Park DY, Lee KC, Yang JW, Kim JS, Kim HJ, Jung JH. Prevalence of the antimicrobial resistance and resistance associated gene in Salmonella spp. isolated from pigs and cattle in slaughterhouse. Korean Journal of Veterinary Service. 2011; 34(1):45-54.
38. Addis Z, Kebede N, Sisay Z, Alemayehu H, Wubetie A, Kassa T. Prevalence and antimicrobial resistance of Salmonella isolated from lactating cows and in contact humans in dairy farms of Addis Ababa: a cross sectional study. BMC Infectious Diseases. 2011; 11(1):222.
39. AKam A, Khelef D, Kaidi R, Rahal KH, Tali-Maamar H, Yabrir B, Laoun A, Mostfaoui A, Boutaiba S, Cozma V. The Frequency of the Shedding of *Cryptosporidium parvum*, F5 spp. in Young Dairy Calves in Mitidja Area (Algeria). Bulletin *Escherichia coli, Rotavirus, Coronavirus* and *Salmonella*of the University of Agricultural Sciences & Veterinary Medicine Cluj-Napoca. Veterinary Medicine. 2011; 68(2).
40. Alemu S, Zewde BM. Prevalence and antimicrobial resistance profiles of *Salmonella enterica* serovars isolated from slaughtered cattle in Bahir Dar, Ethiopia. Tropical Animal Health and Production. 2012; 44(3):595-600.
41. Bolton DJ, O’neill CJ, Fanning S. A preliminary study of *Salmonella, verocytotoxigenic Escherichia coli/Escherichia coli O157* and *Campylobacter* on four mixed farms. Zoonoses and Public Health. 2012; 59(3):217-28.
42. Gorski L, Parker CT, Liang A, Cooley MB, Jay-Russell MT, Gordus AG, Atwill ER, Mandrell RE. Prevalence, distribution and diversity of *Salmonella enterica* in a major produce region of California. Applied and Environmental Microbiology. 2011; 77(8):2734-2748.
43. Sibhat B, Molla Zewde B, Zerihun A, Muckle A, Cole L, Boerlin P, Wilkie E, Perets A, Mistry K, Gebreyes WA. *Salmonella* serovars and antimicrobial resistance profiles in beef cattle, slaughterhouse personnel and slaughterhouse environment in Ethiopia. Zoonoses and Public Health. 2011;58(2):102-9.
44. Alao F, Kester C, Gbagba B, Fakilede F. Comparison of prevalence and antimicrobial sensitivity of *Salmonella* Typhimurium in apparently healthy cattle and goat in Sango-Ota, Nigeria. The Internet Journal of Microbiology. 2012; 10(2).
45. Hiroi M, Kawamori F, Harada T, Sano Y, Miwa N, Sugiyama K, Hara-Kudo Y, Masuda T. Antibiotic resistance in bacterial pathogens from retail raw meats and food-producing animals in Japan. Journal of Food Protection. 2012;75(10):1774-82.
46. Loneragan GH, Thomson DU, McCarthy RM, Webb HE, Daniels AE, Edrington TS, Nisbet DJ, Trojan SJ, Rankin SC, Brashears MM. Salmonella diversity and burden in cows on and culled from dairy farms in the Texas High Plains. Foodborne Pathogens and Disease. 2012; 9(6):549-55.
47. Navarro‐Gonzalez N, Velarde R, Porrero MC, Mentaberre G, Serrano E, Mateos A, Domínguez L, Lavín S. Lack of Evidence of Spill‐Over of *Salmonella enterica* Between Cattle and Sympatric I berian ibex (Capra pyrenaica) from a Protected Area in C atalonia, NE S pain. Transboundary and Emerging Diseases. 2012; 61(4):378-84.
48. Gragg SE, Loneragan GH, Nightingale KK, Brichta-Harhay DM, Ruiz H, Elder JR, Garcia LG, Miller MF, Echeverry A, Porras RG, Brashears MM. Substantial within-animal diversity of Salmonella recovered from lymph nodes, feces and hides of cattle at slaughter. Applied and Environmental Microbiology. 2013; 79(15):4744-4750.
49. Islam MM, Ashrafuzzaman M, Ali MH, Choudhury KA, Khan MS. Characterization, pathogenecity and antibiogram study of *Salmonella* species isolated from apparently healthy and diarrhoeic calves. Intrnational Journal of Bioscience. 2013;3(1):109-20.
50. Kagambèga A, Lienemann T, Aulu L, Traoré AS, Barro N, Siitonen A, Haukka K. Prevalence and characterization of Salmonella enterica from the feces of cattle, poultry, swine and hedgehogs in Burkina Faso and their comparison to human Salmonella isolates. BMC Microbiology. 2013; 13(1):253.
51. Narváez-Bravo C, Rodas-González A, Fuenmayor Y, Flores-Rondon C, Carruyo G, Moreno M, Perozo-Mena A, Hoet AE. *Salmonella* on feces, hides and carcasses in beef slaughter facilities in Venezuela. International Journal of Food Microbiology. 2013; 66(2):226-30.
52. Raufu I, Bortolaia V, Svendsen CA, Ameh JA, Ambali A, Aarestrup FM, Hendriksen RS. The first attempt of an active integrated laboratory‐based *Salmonella* surveillance programme in the north eastern region of Nigeria. Journal of Applied Microbiology. 2013; 115(4):1059-67.
53. Sasaki Y, Murakami M, Haruna M, Maruyama N, Mori T, Ito K, Yamada Y. Prevalence and characterization of foodborne pathogens in dairy cattle in the eastern part of Japan. Journal of Veterinary Medical Science. 2013; 75(4):543-6.
54. Sychanh T, Chaunchom S, Pulsrikarn C, Pornreongwong S, Chaichana P, Boonmar S. *Salmonella* Prevalence in Slaughtered Buffaloes and Cattle in Champasak Province, Lao People’s Democratic Republic.Natural Science. 2013; 47(4):561-570.
55. Wani SA, Hussain I, Beg SA, Rather MA, Kabli ZA, Mir MA, Nishikawa Y. Diarrhoeagenic *Escherichia coli* and *Salmonellae* in calves and lambs in Kashmir: absence, prevalence and antibiogram. Revue Scientifique et Technique(International Office of Epizootics). 2013; 32(3): 833-840.
56. Zare P, Ghorbani-Choboghlo H, Jaberi S, Razzaghi S, Mirzae M, Mafuni K. Occurrence and antimicrobial resistance of *Salmonella spp.* and *Escherichia coli* isolates in apparently healthy slaughtered cattle, sheep and goats in East Azarbaijan province. International Journal of Enteric Pathogens 2014; 2(1):1-4.
57. Zubair AI, Ibrahim KS. Isolation of Salmonella from slaughtered animals and sewage at Zakho abattoir, Kurdistan Region, Iraq. Research Opinions in Animal and Veterinary Sciences. 2013; 3(1):20-4.
58. Bosilevac JM, Gassem MA, Al Sheddy IA, Almaiman SA, Al-Mohizea IS, Alowaimer A, Koohmaraie M. Prevalence *of Escherichia coli O157: H7* and *Salmonella* in camels, cattle, goats, and sheep harvested for meat in Riyadh. Journal of Food Protection. 2015;78(1):89-96.
59. Dong P, Zhu L, Mao Y, Liang R, Niu L, Zhang Y, Li K, Luo X. Prevalence and profile of *Salmonella* from samples along the production line in Chinese beef processing plants. Food Control. 2014; 38:54-60.
60. Al Mawly J, Grinberg A, Prattley D, Moffat J, French N. Prevalence of endemic enteropathogens of calves in New Zealand dairy farms. New Zealand Veterinary Journal. 2015; 63(3):147-52.
61. Barlow RS, McMILLAN KE, Duffy LL, Fegan N, Jordan D, Mellor GE. Prevalence and antimicrobial resistance *of Salmonella* and *Escherichia coli* from Australian cattle populations at slaughter. Journal of Food Protection. 2015; 78(5):912-20.
62. Hailu D, Gelaw A, Molla W, Garedew L, Cole L, Johnson R. Prevalence and antibiotic resistance patterns of *Salmonella* isolates from lactating cows and in-contact humans in dairy farms, Northwest Ethiopia. Journal of Environmental and Occupational Science. 2015; 4(4):171-178.
63. Kuang X, HaihongH, Menghong Dai YulianW, Ijaz A, Zhenli Land Zonghui Y. Serotypes and antimicrobial susceptibility of Salmonella spp. isolated from farm animals in China. Frontiers in Microbiology. 2015; 6:602.
64. Madoroba E, Kapeta D, Gelaw AK. *Salmonella* contamination, serovars and antimicrobial resistance profiles of cattle slaughtered in South Africa. Onderstepoort Journal of Veterinary Research. 2016; 83(1):1-8.
65. Schmidt JW, Agga GE, Bosilevac JM, Brichta-Harhay DM, Shackelford SD, Wang R, Wheeler TL, Arthur TM. Occurrence of antimicrobial-resistant Escherichia coli and *Salmonella enterica* in the beef cattle production and processing continuum. Applied and Environmental Microbiology. 2014;81(2),713-725.
66. Tarazi YH, Abo-Shehada MN. Herd-and individual-level prevalences of and risk factors for *Salmonella* spp. fecal shedding in dairy farms in Al-Dhulail Valley, Jordan. Tropical Animal Health and Production. 2015; 47(7):1241-8.
67. Aboud OA, Adaska JM, Williams DR, Rossitto PV, Champagne JD, Lehenbauer TW, Atwill R, Li X, Aly SS. Epidemiology of *Salmonel*la sp. in California cull dairy cattle: prevalence of fecal shedding and diagnostic accuracy of pooled enriched broth culture of fecal samples. Peer Journal. 2016; 4:e2386.
68. Beyene T, Yibeltie H, Chebo B, Abunna F, Beyi AF, Mammo B, Ayana D, Duguma R. Identification and antimicrobial susceptibility profile *of salmonella* isolated from selected dairy farms, abattoir and humans at Asella town, Ethiopia. Journal of Veterinary Science and Technology. 2016; 7(3):320.
69. El-Gamal AM, EL-Bahi EF. Molecular Characterization of Rectal Carriage of E Coli O157: H7 and Salmonella Spp. in Feedlot Animals and Its Effects on Carcasses Contamination. Alexandria Journal for Veterinary Sciences. 2016; 48(1): 42-49.
70. Eguale T, Engidawork E, Gebreyes WA, Asrat D, Alemayehu H, Medhin G, Johnson RP, Gunn JS. Fecal prevalence, serotype distribution and antimicrobial resistance of *Salmonellae* in dairy cattle in central Ethiopia. BMC Microbiology. 2016; 16(1):20.
71. Hanson DL, Loneragan GH, Brown TR, Nisbet DJ, Hume ME, Edrington TS. Evidence supporting vertical transmission of *Salmonella* in dairy cattle. Epidemiology and Infection. 2016; 144(5):962-7.
