# Supplementary material for: Prevalence and Serotype Diversity of Salmonella in Apparently Healthy Cattle: Systematic Review and Meta-Analysis of Published Studies, 2000–2017
Source: Front Vet Sci. 2019 Apr 9;6:102. doi: 10.3389/fvets.2019.00102 (PMC6476277; doi:10.3389/fvets.2019.00102)
Supplement: Supplementary file 2 [file Table_2.docx]

Supplemental Table 2: List and frequency of *Salmonella* serotypes categorized as “others” in the text

| **Africa** | | **Asia** | | **Australia** | | **North America** | |
| --- | --- | --- | --- | --- | --- | --- | --- |
| Aberdeen | 2 | Blockley | 1 | Aberdeen | 2 | Newington | 19 |
| Albany | 2 | Brunei | 1 | Give | 2 | Cubana | 15 |
| Anatum | 2 | Calabar | 1 | Meleagridis | 2 | Soerenga | 15 |
| Apeyeme | 2 | Kentucky | 1 | Montevideo | 2 | Thompson | 13 |
| Chandans | 2 | Lagos | 1 | Seftenberg | 2 | Ohio | 12 |
| Colobane | 2 | O10/HMC | 1 | Amsterdam var 15+ | 1 | Dublin | 11 |
| Dahra | 2 | Stanley | 1 | Bootle | 1 | Tennessee | 9 |
| Eastbourne | 2 | Tumodi | 1 | Chailey | 1 | Livingstone | 8 |
| Heidelberg | 2 | Weltevreden | 1 | Enteritidis | 1 | Oranienberg | 7 |
| Infantis | 2 | Havana | 1 | Heidelberg | 1 | Albany | 6 |
| Kalina | 2 |  |  | Mgulani | 1 | Reading | 6 |
| Kingston | 2 |  |  | Newport | 1 | Cholerasuis | 5 |
| Kokomlemle | 2 |  |  | Potsdam | 1 | Barranquilla | 4 |
| Korlebu | 2 |  |  | Rubislaw | 1 | Idikan | 4 |
| Nottingham | 2 |  |  | Subsp I 16: I, v: - | 1 | lille | 4 |
| Othmarschen | 2 |  |  | Subsp I 3, 10: -: - | 1 | SanDiego | 3 |
| Poona | 2 |  |  | Victoria | 1 | 3,10:1, monophasic | 2 |
| Waycross | 2 |  |  | Virchow | 1 | 4,12:2, monophasic | 2 |
| Abaetetuba | 1 |  |  | Wangata | 1 | Bredeney | 2 |
| Antwepen | 1 |  |  |  |  | Rough O: b:1,2 | 2 |
| Banana | 1 |  |  |  |  | Bardo | 1 |
| Bareilly | 1 |  |  |  |  | Bergen | 1 |
| Bargny | 1 |  |  |  |  | Cambridge | 1 |
| Brancaster | 1 |  |  |  |  | Denver | 1 |
| Brive | 1 |  |  |  |  | Hartford | 1 |
| Carmel | 1 |  |  |  |  | Kedougou | 1 |
| Carno | 1 |  |  |  |  | Kiambu | 1 |
| Chester | 1 |  |  |  |  | Rough | 1 |
| Colindale | 1 |  |  |  |  | Schwarzengrund | 1 |
| Dakar | 1 |  |  |  |  | Uganda | 1 |
| Frintrop | 1 |  |  |  |  | havana | 1 |
| Gokul | 1 |  |  |  |  | litchfield | 1 |
| Haifa | 1 |  |  |  |  | Minnesota | 30 |
| Hayindongo | 1 |  |  |  |  |  |  |
| I:6,7,14: –: I, w | 1 |  |  |  |  |  |  |
| II 40: b: - | 1 |  |  |  |  |  |  |
| Ikeja | 1 |  |  |  |  |  |  |
| Ilala | 1 |  |  |  |  |  |  |
| Kalamu | 1 |  |  |  |  |  |  |
| Livingstone | 1 |  |  |  |  |  |  |
| Mikawasima | 1 |  |  |  |  |  |  |
| Moero | 1 |  |  |  |  |  |  |
| Monschaui | 1 |  |  |  |  |  |  |
| Montevideo | 1 |  |  |  |  |  |  |
| Oranienberg | 1 |  |  |  |  |  |  |
| Rissen | 1 |  |  |  |  |  |  |
| S. group B 4,5,12: -: - | 1 |  |  |  |  |  |  |
| S. group C 6,7,14: d: - | 1 |  |  |  |  |  |  |
| S. group E 3,10: e, h: - | 1 |  |  |  |  |  |  |
| Salford | 1 |  |  |  |  |  |  |
| Schwarzengrund | 1 |  |  |  |  |  |  |
| Trachau | 1 |  |  |  |  |  |  |
| Umbadah | 1 |  |  |  |  |  |  |
| Yoruba | 1 |  |  |  |  |  |  |
